# Tapping our resources: do preoperative aspirations add diagnostic value in hip and knee periprosthetic joint infection?

**DOI:** 10.5194/jbji-11-315-2026

**Published:** 2026-06-03

**Authors:** Anne Spichler-Moffarah, Lauren Daddi, Ilda Molloy, Tyler Luu, Duc Nguyen, Marjorie Golden

**Affiliations:** 1 Division of Infectious Diseases, Department of Internal Medicine, Yale School of Medicine, New Haven, CT, USA; 2 Yale School of Medicine, New Haven, CT, USA; 3 Department of Orthopedics & Rehabilitation, Division of Adult Reconstruction, Yale School of Medicine, New Haven, CT, USA; 4 Department of Infectious Diseases, Yale School of Medicine, 200 Orchard Street, New Haven, CT, USA

## Abstract

**Background**: Discrepancies exist between preoperative synovial aspirations and intraoperative cultures. It is unclear whether the results of preoperative arthrocentesis add diagnostic value for patients with possible prosthetic joint infection (PJI). **Methods**: We retrospectively identified adult patients treated surgically for hip and knee PJI who underwent both preoperative aspiration and intraoperative sampling. Synovial and intraoperative culture results were analyzed. Cultures were defined as concordant if they yielded the same organism(s) or if both were negative. Cultures were considered to be discordant if different organisms were isolated or if one had growth and the other was sterile. Demographic, clinical, and laboratory variables between groups were compared. **Results**: Among 75 patients, 54 (72 %) had concordant results, and 21 (28 %) had discordant results. In the discordant group, 7 of 21 patients (33 %) had negative synovial cultures with positive intraoperative cultures. Among culture-positive concordant patients, staphylococci and streptococci were the most common isolates, with 20 % gram negatives. True culture-negative PJI was uncommon ( n=1 ). Preoperative antibiotic exposure was frequent ( >50 %) in both groups. **Conclusion**: In a cohort of hip and knee PJI patients treated surgically, preoperative synovial aspiration cultures were concordant with intraoperative cultures in 72 % of cases, but negative synovial aspirations did not reliably exclude infection. Given the observed discordance, our findings support the obtainment of preoperative synovial aspiration and the collection of multiple intraoperative specimens at the time of surgery.

## 1 Introduction

Identification of causative microorganisms guides antimicrobial therapy in patients with prosthetic joint infections (PJIs) (Tande and Patel, 2014; Nelson et al., 2023). Organisms causing PJI can be identified through samples such as synovial fluid aspiration or tissue culture. These methods differ in terms of sensitivity, but both support a diagnosis of PJI (Boyle et al., 2021; Akkaya et al., 2023). Results of preoperative arthrocentesis may fulfill diagnostic criteria and establish a microbiologic diagnosis, even before operative debridement is performed (Tande and Patel, 2014; Patel, 2023; Akkaya et al., 2023). Often, the pathogen identified in preoperative synovial fluid guides surgical treatment decisions, but it is unknown whether this test has the same diagnostic yield as intraoperative tissue cultures (Boyle et al., 2021). Prior studies demonstrate concordance rates of 52 % to 78 % (Akkaya et al., 2023; Boyle et al., 2021) between preoperative aspiration results and intraoperative tissue cultures.

Antibiotic exposure before surgery in patients with positive preoperative aspirations may affect intraoperative culture yield and influence concordance with aspiration results. However, in real-world practice, initiation of antibiotics cannot always be deferred, particularly in patients with systemic illness or concerns regarding sepsis. Discordant organisms have been identified in preoperative aspirates as compared with intraoperative specimens even in the absence of prior antibiotic therapy (Boyle et al., 2021; Akkaya et al., 2023).

Our aim was to evaluate concordance between preoperative synovial aspiration and intraoperative cultures in patients undergoing surgery for hip and knee PJI.

## 2 Materials and methods

### 2.1 Ethics

This study was approved by the Yale University Institutional Review Board.

### 2.2 Study design

This was a retrospective review of adult patients admitted to an urban tertiary academic center from September 2017 to December 2020 with a first episode of hip or knee PJI. A first episode was defined as the first documented episode of PJI in the index joint during the study period. Inclusion criteria specified subjects ≥ 18 years of age who underwent surgical management for PJI and had both preoperative synovial aspirations and intraoperative cultures performed. We included patients with symptoms of both acute and chronic infection. Patients managed exclusively as outpatients, transferred from other institutions, managed with non-surgical interventions, or with previous PJIs were excluded. We excluded patients whose only positive culture came from sonication of hardware as this was not used for all patients. Subjects were screened and identified using ICD 10 codes for PJI of the knee or hip by the Yale–New Haven Hospital Joint Data Analytics (JDAT) team.

### 2.3 Data collection

Chart reviews were performed by two infectious-disease physicians in consultation with an orthopedic surgeon. Abstracted variables included the following: demographics, clinical presentation, inflammatory markers on admission, synovial aspiration results, microbiology (synovial aspiration and intraoperative cultures), and type and duration of antibiotics given in relation to preoperative arthrocentesis and surgery.

## 3 Definitions

Patients were considered to have PJI if they met the criteria outlined in the 2018 International Consensus Meeting (ICM18) (Parvizi et al., 2018). We applied IDSA criteria to define late (within 3 months of arthroplasty), delayed (3–24 months postoperative), or late ( >24 months postoperative) (Osmon et al., 2013) PJI.

Concordance was defined by the growth of identical organism(s) on preoperative aspiration and operative specimens. Patients were also considered to have concordant results if neither specimen grew any microorganisms. Results were discordant if different organisms grew from preoperative aspirations compared with intraoperative cultures or if one specimen grew an organism and the other was sterile.

In our institution, synovial and tissue specimens are incubated for 5 d unless *Cutibacterium acnes* (*C. acnes*) culture is requested. *C. acnes* cultures are routinely held for 14 d. Specimens are not routinely inoculated into blood culture bottles. Our diagnostic algorithm recommends taking four to five samples of intraoperative specimens per patient, as per ICM recommendations (Parvizi et al., 2018).

The type of surgical management was based on the surgeon's clinical judgment and was described as debridement, antibiotic, and implant retention (DAIR); one stage; two stage; or amputation.

### Statistics

Data analysis and the creation of figures were performed in RStudio using R (v4.5.1). Chi-squared and Wilcoxon rank sum tests were used to evaluate significant differences in terms of clinical variables between concordance and discordance groups. Significance was defined as a p value of less than 0.05.

## 4 Results

A total of 75 patients met the inclusion criteria (mean age of 69.7±13.3 years, 50.7 % female, 53 % white). A total of 54 (72 %) patients showed concordance and 21 patients (28 %) showed discordance between synovial aspiration and intraoperative cultures (Table 1). Demographics, clinical manifestations, and surgical management for PJI are shown in Table 1. The time from synovial aspiration to intraoperative cultures and/or surgery was significantly different between groups ( p=0.002 ), with a median of 1 d in the concordant group and 4 d in the discordant group (Table 1).

**Table 1:** Demographic and clinical characteristics for discordant and concordant groups. Bold values mean statistic significant.

	Discordance	Concordance	p value^*^
	[N=21]	[N=54]	
Age (years), mean ± SD	73.9±13.8	68.4±12.8	0.091
Female sex (%)	14 (66.7)	23 (42.6)	0.11
Race			0.079
White (%)	18 (85.7)	35 (64.8)	
Black (%)	1 (4.8)	14 (25.9)	
Not listed (%)	2 (9.5)	5 (9.3)	
Ethnicity			1
Hispanic (%)	1 (4.8)	3 (5.6)	
Non-Hispanic (%)	20 (95.2)	51 (94.4)	
BMI, mean ± SD	31.3±6.4	29.6±6.6	0.249
Joint			0.828
Hip (%)	5 (23.8)	16 (29.6)	
Knee (%)	16 (76.2)	38 (70.4)	
Type of Surgery			0.54
DAIR (%)	13 (61.9)	32 59.3)	
Revision (%)	3 (14.3)	4 (7.4)	
Spacer (%)	5 (23.8)	18 (33.3)	
Time from arthroplasty to diagnosis			0.493
Early ( <3 months)	18 (85.7)	13 (24.0)	
Delayed ( ≥3 –24 months)	3 (14.3)	12 (22.2)	
Late ( >24 months)	0	29 (53.7)	
Time from symptoms to diagnosis, mean (IQR)	9 (16)	7.5 (11)	0.782
Acute ( ≤21 d) (%)	18 (85.7)	43 (79.6)	
Chronic ( >21 d) (%)	3 (14.3)	11 (20.4)	
Synovial WBC, median (IQR)	27 000 (51 000)	62 700 (84 295)	**0.006**
CRP, median (IQR)	132.4 (160.5)	177 (154)	0.355
ESR, median (IQR)	66.5 (53.5)	74 (53)	0.949
Reason for discordance			
[-] synovial and [+] OR (%)	7 (33.3)	0	
[+] synovial and [-] OR (%)	11 (52.4)	0	
[+] with different organisms (%)	3 (14.3)	0	
Reason for concordance			
[-] synovial and culture (%)	0	9 16.7)	
[+] with same organisms (%)	0	45 (83.3)	
Aspiration to surgery time (d), median (IQR)	4 (5)	1 1.75)	**0.002**
Total intraoperative cultures, median (IQR)	3 (1)	3 (2)	0.6105
Positive intraoperative cultures, median (IQR)	0 (3)	2 (3)	**0.0287**
Pre-aspiration antibiotics (%)	5 (23.8)	17 (31.5)	0.709
Preoperative antibiotics (%)	10 (47.6)	37 (68.5)	0.157

^*^ Since not all subjects receive antibiotics (either preoperative or pre-aspiration), individual antibiotic percentages will not sum to 100. Some patients received both pre-aspiration and preoperative antibiotics. All patients who received pre-aspiration antibiotics also received preoperative antibiotics. OR denotes operating room.

**Figure 1:**
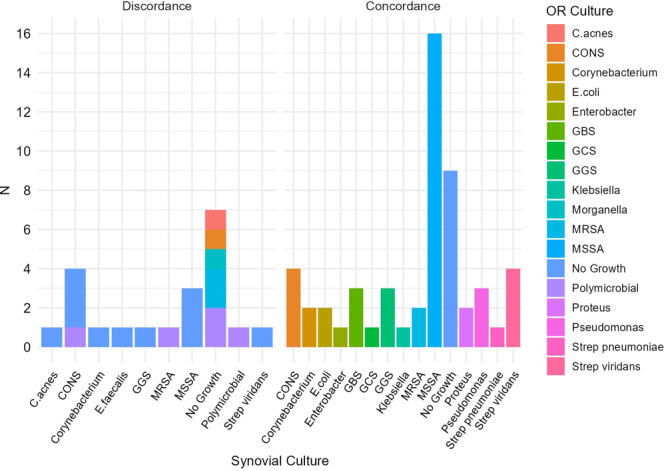
Microorganisms cultured and characterization of culture-negative infections. Microorganisms identified in concordance and discordance groups, comparing synovial and intraoperative (OR) culture results.

Most patients had an average of three and a half intraoperative specimens (range of one to nine). Methicillin-susceptible *Staphylococcus aureus* (MSSA) was the most common organism identified, followed by coagulase-negative Staphylococci (CoNS), with gram-negative and *Streptococcus* species occurring in similar frequency (Fig. 1). Polymicrobial infection was identified in five (6.7 %) intraoperative cultures and one (1.3 %) synovial culture. Of the five polymicrobial intraoperative cultures, discordance with synovial cultures occurred for three reasons: (1) at least one differing organism (two out of five), (2) negative synovial culture with polymicrobial intraoperative growth (two out of five), or (3) polymicrobial growth in both samples but with entirely different organisms (one out of five).

Among culture-positive concordant patients (45 out of 54, 83 %), isolates included MSSA ( n=16 , 35 %), CoNS ( n=4 , 1 %), *Streptococcus* species ( n=12 , 26.7 %), gram-negative organisms ( n=9 , 20 %), and others (Fig. 1). A total of 9 of 54 patients (16.7 %) had negative synovial and intraoperative cultures (Fig. 1).

Among the 54 concordant patients with both cultures being positive, the majority (92.6 %) had received antibiotics before either aspiration or surgery. In the discordant group, 7 of 21 patients (33 %) had negative synovial (no growth) cultures but positive intraoperative cultures, with no antibiotics prior to synovial aspiration (Fig. 1). The remaining 14 out of 21 discordant patients (67 %) had positive preoperative synovial cultures: 11 (78 %) had negative intraoperative cultures (no growth), and 3 (22 %) had growth in both synovial and intraoperative specimens but with different organisms identified (Table 1). As expected, based on the group definitions, a higher proportion of patients in the concordant group had positive intraoperative cultures since 11 out of 21 discordant cases were classified as discordant specifically due to negative intraoperative cultures.

There was no substantial clinical difference in terms of fever ( p=1 ) and sinus tract ( p=0.86 ) between concordant and discordant cases. We did have more patients with knee PJI, but hip involvement was comparable between groups (29.6 % vs. 23.8 %) ( p=0.83 ), and most patients in both cohorts underwent DAIR (59.3 % vs. 61.9 %) ( p=0.54 ). The concordant group had a greater proportion of late infections ( >24 months; 53.7 % vs. 0 %), while early infections ( <3 months) were more common in the discordant group (85.7 % vs. 24 %), though these differences were not statistically significant ( p=0.49 ). The discordance group had a significantly higher time between the aspiration and surgery ( p=0.002 ).

Among the 18 patients with bacteremia, 12 (66.7 %) had concordance between the blood culture isolate and the intraoperative culture, and 16 (88.9 %) had concordance between the blood culture isolate and the preoperative synovial fluid culture. For 14 out of 18 (77.8 %), there was concordance between intraoperative tissue culture and preoperative synovial fluid culture. Bacteremia was more frequent in the concordant group (25.9 % vs. 19.0 %); however, this did not achieve statistical significance ( p=0.75 ). Bacteremia was most often due to MSSA and was predominantly associated with late infection.

Use of antibiotics was higher in the concordant vs. discordant group: both prior to arthrocentesis (31.5 % vs. 23.8 %) ( p=0.71 ) and prior to surgery (68.5 % vs. 47.6 %) ( p=0.16 ). However, the difference did not reach statistical significance. For the 20 patients receiving pre-aspiration antibiotics, the average duration of therapy was 4.2 d, with a range of 1–14 d. For the 47 patients who received preoperative antibiotics, the average duration was 4.9 d, with a range of 1–34 d. Antibiotic agents were as follows for the concordant and discordant groups, respectively: Vancomycin – 29 (53.7 %) and 7 (33 %); piperacillin-tazobactam – 13 (24.1 %) and 4 (19 %); ceftriaxone – 8 (14.8 %) and 1 (4.8 %); cefazolin – 4 (7.4 %) and none; and ceftazidime – 4 (7.4 %); others, such as oxacillin, cefepime, Bactrim, and ertapenem, were prescribed in small numbers.

## 5 Discussion

In this 75-patient cohort with hip and knee PJI, we found a concordance between preoperative synovial fluid and intraoperative cultures of 72 %. The concordance rate suggests that preoperative aspiration cultures frequently identify the same organism later recovered intraoperatively and can provide early microbiologic information that may implicate surgical decision-making. Discordance, however, remained common (28 %), underscoring that aspiration results cannot be relied upon alone, particularly to rule out infection or to replace a collection of multiple intraoperative specimens. MSSA was the most frequently isolated organism, and true culture-negative PJI was uncommon. Notably, discordance was seen more often in early infections, whereas late infections tended to show concordant results.

The interval between aspiration and surgery was significantly longer in the discordant group, although the absolute difference was modest. This may represent an important confounder. A longer interval could allow for additional antibiotic exposure, changes in bacterial burden, or differences in sampling yield between the preoperative and intraoperative time points. Alternatively, patients with more clinically apparent infections or more virulent organisms may have proceeded to surgery more urgently, which could contribute to higher concordance in those cases. Because of the retrospective design and small sample size, we cannot determine whether the time from aspiration to surgery independently contributed to discordance.

Prior studies have assessed the predictive value of preoperative synovial and intraoperative cultures (Boyle et al., 2021; Akkaya et al., 2023; Schulz et al., 2021; Matter-Parrat et al., 2017), and some question whether intraoperative cultures are necessary with positive synovial cultures (Li et al., 2021). This issue is relevant across acute and chronic, as well as polymicrobial and monomicrobial, PJI. In chronic PJI, synovial aspiration may be less frequently positive due to lower organism burden within biofilm and the predominance of less virulent organisms, and, in this situation, intraoperative cultures have been shown to yield more polymicrobial growth than preoperative aspirates (Schulz et al., 2021; Matter-Parrat et al., 2017). In our cohort, we observed high concordance in late infections in comparison with early infections and low rates of polymicrobial growth. While this finding differs from what has been shown by other groups, it may reflect our low rates ( <20 %) of chronic infections (which usually are associated with late infection), small numbers, or low inoculum in the setting of polymicrobial infections.

We found 72 % concordance between preoperative aspiration and intraoperative tissue cultures. Infections were primarily monomicrobial, led by MSSA, followed by CoNS and *Streptococcus* species. While Li et al. (2021) reported greater discordance with *Streptococcus* (Li et al., 2021), our small sample size study showed high concordance, limiting firm conclusion. We had low rates of culture-negative infection, with only one patient meeting criteria for true culture-negative PJI. This contrasts with prior reports showing culture-negative rates of 18 % for intraoperative samples and 28 % for preoperative aspirates in knee PJI (Akkaya et al., 2023), which may reflect institutional microbiologic methods or differences in patient selection. We do not have data on the volume of fluid obtained at the time of arthrocentesis, which may impact the yield of culture. This study focused on comparing culture results from preoperative aspirations and intraoperative cultures, and so we did not compare cell counts between the two specimens.

Inadequate intraoperative sampling (less than three specimens) increases the risk of false-negative intraoperative cultures and may therefore overestimate discordance in cases where synovial cultures were positive but intraoperative cultures were negative. We did have an average of three and a half samples in our cohort, close to the current recommendations (Osmon et al., 2013; McNally et al., 2021); therefore, this does not change the main interpretation that negative preoperative synovial cultures do not exclude infection and that obtaining multiple intraoperative specimens remains essential. In addition, we do not routinely send either preoperative or intraoperative specimens for broad-range PCR testing.

Our findings align more closely with Boyle et al., who reported 76.8 % concordance and similar rates in hip and knee arthroplasties (Boyle et al., 2021). Unlike their study – which included only revision procedures meeting ICM 2018 criteria – our cohort reflects routine clinical practice by including all patients suspected of PJI across diagnostic criteria and surgical approaches, with nearly all cases having multiple intraoperative tissue cultures, minimizing sampling bias.

In another study, patients with PJI diagnosed by ICM 2018 were required to have a 2-week antibiotic holiday prior to arthrocentesis. In addition, no antibiotics were administrated before obtaining intraoperative tissue specimens (Li et al., 2021). This strategy may not reflect general practice where patients with fever, cellulitis, or signs of sepsis may require empiric antibiotic therapy. A total of 37 out of 54 (68.5 %) patients in the concordance group received antibiotics; among the nine culture-negative patients, almost half (44.4 %) had received antibiotics. We did not exclude patients who received antibiotics prior to arthrocentesis or surgery. Despite antibiotics often being given prior to culture, our concordance rate still approached 75 %. Patients with late infections had a high rate of concordance and higher rates of bacteremia, which could suggest a higher organism burden in the joint. Patients with late acute infection may have less time to develop extensive biofilm, which may translate into higher results of positive preoperative aspirations.

Clinically, these findings support a dual-sampling strategy. Preoperative aspiration can provide actionable microbiologic information before surgery and may help guide antimicrobial planning, surgical approach, and counseling. However, given the observed discordance, particularly cases with negative synovial cultures and positive intraoperative cultures, aspiration culture should not be viewed as definitive in isolation. When clinical suspicion for PJI remains high, surgeons should continue to obtain multiple intraoperative cultures regardless of the preoperative aspiration results.

The limitations of our study include its retrospective, single-center design and relatively small sample size, which limited subgroup analyses according to the causative organism, joint involved, surgical management, antibiotic exposure, and antibiotic type. The small number of discordant cases also prevented adjusted analysis, and so unrecognized confounding may have influenced our findings, including differences in sex distribution, hip versus knee involvement, timing from aspiration to surgery, and clinical severity among patients who received antibiotics before aspiration. Antibiotic exposure before aspiration and/or surgery may have reduced culture yield, although this also reflects real-world management of patients with systemic illness, cellulitis, bacteremia, or concern for sepsis. Microbiologic methods may also have influenced concordance: five discordant cases had fewer than three intraoperative specimens submitted for culture; our cohort had a median of three intraoperative specimens, which is below current ICM recommendations of four to six samples for suspected hip or knee PJI; specimens were not routinely inoculated into blood culture bottles; and molecular testing was not routinely performed, all of which may have reduced culture yield, particularly in low-inoculum infections, increasing the risk of false-negative cultures and apparent discordance. Some discordant cases, especially those with positive synovial cultures and negative intraoperative cultures, may reflect false-negative intraoperative cultures rather than true microbiologic discordance. Finally, synovial leukocyte count and differential and aspiration fluid volume were not systematically available, limiting the interpretation of our findings to culture concordance rather than the full diagnostic performance of synovial fluid testing.

Ultimately, 72 % of patients in our study had concordance between preoperative aspiration and intraoperative cultures, with few culture-negative patients and a predominance of MSSA among concordant cases. Although overall concordance was high, discordance remained clinically meaningful, including cases with negative preoperative synovial cultures but positive intraoperative cultures. Therefore, negative preoperative aspiration cultures should not be used to exclude PJI when clinical suspicion remains high. Given the importance of identifying the causative pathogens in guiding antimicrobial therapy and surgical decision-making, our findings support a dual-sampling strategy: obtaining preoperative aspiration when feasible while continuing to collect multiple intraoperative specimens at the time of surgery.

## Data Availability

The code and data used in this work are available from the corresponding author upon request.
